# A Patient with Recurrent Strokes: Approach to Coagulopathy

**DOI:** 10.1055/a-2161-1262

**Published:** 2023-09-27

**Authors:** Gabriel Alejandro Zúñiga, Pranav Kandula, Hardy Sandefur, Alfonso J. Tafur

**Affiliations:** 1 School of Medicine, Universidad Católica de Santiago de Guayaquil, Guayaquil, Ecuador; 2 Division of Cardiology, NorthShore University HealthSystem, Evanston, Illinois, United States; 3 College of Engineering, Ohio State University, Columbus, Ohio, United States

**Keywords:** anticoagulation, venous thromboembolism, antiphospholipid antibody syndrome, recurrent strokes

## Abstract

Despite anticoagulation recommendations, patients may present with recurrent events. While medication adherence is always a concern, assessment of anticoagulation failure demands a systematic approach, taking into account the potential limitations of anticoagulants and a review of differential diagnoses for comorbidities. We illustrate our approach in a case presentation.

## Introduction

Anticoagulation medications are a preventive treatment which are indicated in a plethora of cardiovascular conditions, including venous thromboembolism (VTE), atrial fibrillation, myocardial infarction, and ischemic stroke. Selecting the correct anticoagulant, including dose, route, monitoring, and method of action, is imperative to the mitigation of bleeding and thromboembolic complications. Anticoagulation failure can occur and may lead to cardiovascular outcomes, recurrence of thromboses, or even mortality. Long-term anticoagulation failure can occur either with vitamin K antagonists (VKAs) 1 or direct oral anticoagulants. 2

We present a patient with underlying coagulopathy to illustrate challenges in anticoagulant selection and monitoring.

## Case Presentation

A 64-year-old male presented to the emergency department with altered mental status, headache, and dizziness. Past medical history included antiphospholipid antibody syndrome (APS), anticardiolipin antibody (ACA) positivity, chronic kidney disease, epilepsy, and recurrent ischemic strokes well controlled with warfarin. He was a former smoker (0.5 pack/year) and has not smoked for the last 40 years. The patient's most recent stroke was about 20 years prior, and he had no family history for recurrent strokes. Wife reported excellent adherence to his warfarin therapy and he had been attending the monitoring clinic on schedule. His head computed tomography (CT) with contrast revealed small areas of well-circumscribed low attenuation in the cerebellar hemispheres bilaterally that were compatible with previous ischemic changes, and magnetic resonance imaging (MRI) of the brain revealed small foci of restricted diffusion in the corpus callosum, left consulate gyrus, left thalamus, and right parietal lobe that were consistent with acute or recent infarcts. Angio-CT of the head and neck showed a short segment narrowing of the distal V4 segment of the left vertebral artery which was unchanged from previous evaluations and minimal calcification of the right cervical internal carotid artery without significant stenosis. Angio-CT of the chest showed no indications of narrowing, blockage, aneurysm, or plaque accumulation in the pulmonary vessels or the aorta. Echocardiography was unremarkable except for a mild tricuspid valve regurgitation and no thrombus was found.

**Fig. 1:**
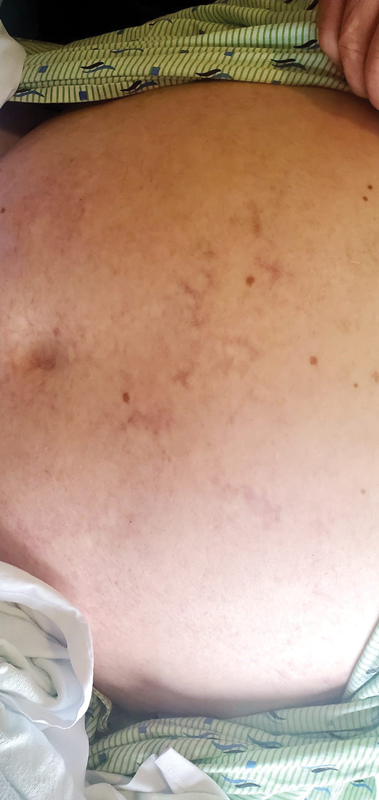
Lesion found on the abdominal area of the patient.

**Fig. 2:**
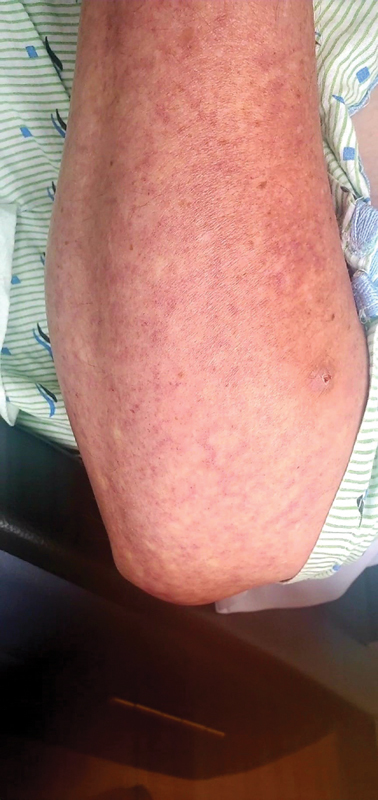
Lesion found on the forearm of the patient.

The initial emergency department physical examination revealed a confused, morbidly obese patient (body mass index: 48) with no paresthesia. His pulses were present from head to toes and no bruits were auscultated in the carotid and subclavian arteries. Vital signs were within normal range. There were no arrhythmias or ST changes on the electrocardiogram. The patient denied sweating, fatigue, chest pain, dyspnea, bruising, hemoptysis, epistaxis, hematemesis, hematochezia, and hematuria. The patient, although he was not hypertensive or diabetic, was a morbidly obese, which alone is a risk factor for accelerated atherosclerosis that could eventually lead to brain ischemic disease. Imaging and additional tests were done in order to assess this risk factor as a probable cause of the ischemic disease, but it was not the case. The following lesions were observed on his trunk and extremities ( Figs. 1 and 2 ). When asked about the lesions, he denied pain and previous exposure to heat or burns in the area. However, he did endorse that the lesions had been there for many years, but were not present at birth. Additionally, he noted that the lesions were not sensitive to warm or cold temperatures.

1. Which of the following is the most likely identification of lesions presented above?
  - Cutis marmorata
  - Erythema ab igne
  - Livedo racemosa
  - Erythrocyanosis
Erythema ab igne is usually caused by exposure to heat that is used to relieve localized pain or cold. The lesions often appear as net-like erythematous and hyperpigmented skin lesions. The size and shape of the lesions are typically related to the heat source. 3 Our patient did not have an exposure to heat in the area of the lesion, thus making this diagnosis unlikely.
Erythrocyanosis is a persistent, dusky erythema occurring at sites with a thick layer of underlying subcutaneous fat, like in the lower extremities. It is exacerbated by cold. 4 The appearance and distribution of the lesion, and its indifference to cold climate make this diagnosis unlikely.
Cutis marmorata telangiectatica congenita is an uncommon congenital capillary vascular malformation that is characterized by fixed patches of mottled skin with a net-like or reticulate blue patches. 5 It is usually associated with congenital abnormalities and present at birth. This is an unlikely diagnosis for this patient.
Livedo racemosa (LRa) is a cutaneous finding characterized by a persistent, erythematous, or violaceous discoloration of the skin, in a broken, branched, discontinuous, and irregular pattern. 6 Some authors make a distinction between two forms of livedo: livedo reticularis, which consists of a complete lace pattern, 7 and LRa. In this case, the description of the lesion is most compatible with LRa, making it the most likely diagnosis.
LRa is always secondary to organic disorders. Thrombotic processes may be involved which lead the clinicians to search for possible systemic vascular diseases, like the recurrent ischemic strokes in this patient. Given the findings of previously known chronic ischemic changes with no intracranial bleeding on his CT, several areas of restricted diffusion consistent with recent infarcts scattered among both hemispheres on his MRI, and labs which revealed thrombocytopenia, anemia, and creatinine elevation lead us to conclude that this patient had a significant vascular disease. See Table 1 for labs.
2. Based on the patient's clinical history and the physical exam findings, which of the following is the most likely diagnosis for this patient?
  - Divry van Bogaert syndrome
  - Deficiency of adenosine deaminase 2
  - Moyamoya angiopathy
  - Sneddon syndrome
Divry van Bogaert syndrome (DVB) is a rare disease characterized by juvenile-onset cognitive impairment, diffuse white matter hyperintensities, and the presence of LRa. The clinical course for DVB is severe and progressive, often leading to dementia. 8 DVB has a hereditary trait and a characteristic angiomatosis on cerebral angiography. Given the lack of juvenile-onset cognitive impairment and no family history of these findings, DVB is unlikely in this case.
Deficiency of adenosine deaminase 2 (DADA2) is a monogenic vasculitis syndrome caused by mutations in the adenosine deaminase 2 (ADA2) gene. The onset of the disease is usually in childhood. The major clinical feature is vasculopathy/vasculitis of small- and medium-sized arteries. Around 50% of patients have presenting symptoms of LRa, stroke, and fever. 9 The diagnostic gold standard for DADA2 is the detection of ADA2 gene mutations. The onset of presentation of DADA2 and the lack of ADA2 mutations make this diagnosis unlikely for this case.
Moyamoya angiopathy (MA) is a rare cerebrovascular disorder characterized by a progressive stenosis of the terminal portion of the internal carotid arteries and the development of fragile abnormal collateral vessels leading to ischemic and hemorrhagic cerebrovascular events. 10 It is more common in the Asian population and most commonly affects children but adults can also be affected. In adults, symptoms may occur between 30 and 50 years of age. LRa has been found in more than 12% of patients with MA. 11 Cerebral angiography is used to make a diagnosis, although MRI and CT can also help in some cases. The odd presentation plus the lack of defining characteristics of MA in the patient's imaging make this choice less likely.
Sneddon syndrome (SS) is a rare medium vessel vasculopathy which characteristically presents with LRa and neurological symptoms including headaches, vertigo, transient ischemic attacks, stroke, and seizures. The cause is typically unknown but it can be associated with autoimmune diseases; it is not uncommon for SS to co-exist with APS. APS is an acquired thrombophilia, defined by the occurrence of thrombosis or pregnancy morbidity in the presence of persistently positive antiphospholipid (aPL) antibodies. 12 Approximately 80% of SS patients have an aPL antibody marker. These antibodies may play a pathogenetic role in some cases of SS, and have subtle but different characteristics between them. 13 14 One of the diagnostic hallmarks of SS is Lra, which usually presents early and may precede the onset of stroke by years. 15 With this background in mind, SS was the most likely diagnosis. SS can be a diagnostic challenge, as it is primarily a clinical diagnosis, without definitive diagnostic testing. However, in SS, brain imaging usually shows cerebral infarcts or hemorrhages in multiple arterial territories with white matter abnormalities. 16 Skin biopsy has also been suggested as a diagnostic tool, although abnormalities in skin biopsies are not specific for SS and should always be interpreted within the appropriate clinical context. Nevertheless, skin biopsies have been shown to be helpful in diagnosing SS. 17 Notably, Lra is an important prerequisite for the diagnosis of SS.
3. What would be the most common finding in the histopathology of the biopsy of the lesion in this patient?
  - Proliferation of smooth muscle cells of the tunica media and occlusion of the lumen of the small- and medium-sized arteries of the skin.
  - Dense lymphocytic inflammatory infiltrates in the muscular vessel wall, affecting the small- and medium-sized arteries of the dermis and subcutis.
  - Predominant interstitial neutrophil and macrophage infiltration with perivascular T-lymphocytes.
  - Hyalinization, thickened blood-vessel walls, fibrin deposition, vascular occlusion by thrombosis, and minimal inflammation.
Dense lymphocytic inflammatory infiltrates can be found in lymphocytic thrombophilic arteritis, a primary lymphocytic vasculitis that presents with LRa or macular hyperpigmentation. 18 Interstitial neutrophil and macrophage infiltration is commonly found in DADA2, 9 as previously described in this article, but hyalinization, thickened blood-vessel walls, fibrin deposition, vascular occlusion by thrombosis, and minimal inflammation are typical of livedoid vasculopathy, a rare chronic vascular disorder characterized by persistent painful ulceration of the lower extremities. 19
Proliferation of smooth muscle cells of the tunica media and occlusion of the lumen of the small- and medium-sized arteries of the skin are the usual findings in biopsies of LRa in SS. 17
On day 3 of the patient's hospitalization, vascular medicine was consulted. Because anticoagulation failure was suspected, additional laboratory tests were ordered. ACA and anti-beta 2 glycoprotein 1 (B2GPI) antibodies were reassessed, of which only ACA was positive. An elevated international normalized ratio (INR: 5.7) and prothrombin time (PT: 26 seconds) were noted. Target INR for this patient was 3.5 to 4.5, and his time in therapeutic range (TTR) was calculated using the Rosendaal method 20 and was 75% of a total of 30 days. Aside from the intrapatient variability of the INR attributed to food, drug interactions, and patient nonadherence, the INR of patients with aPL antibodies can be falsely elevated. For this reason, there are times when we cannot assess the efficacy of warfarin with PT/INR. Factor II or factor X quantitation can be helpful when INR is unreliable; chromogenic assays are more reliable than clot-based assays in patients with APS. Factor II was not in therapeutic level (52%), suggesting that INR was an unreliable test to monitor warfarin anticoagulation in this case.
4. What alternatives to anticoagulation would you consider given the mismatch between factor II and the INR of this patient?
  - Unfractionated heparin 250 U/kg/12 h SC
  - Direct oral anticoagulants
  - Enoxaparin 120 mg/day SC
  - Fondaparinux 7.5 mg/day SC
The patient's weight (140 kg) complicated the dosing for unfractionated heparin. For the obese and morbidly obese patient, there is an emphasis on striking a balance between achieving effective anticoagulation but avoiding bleeding. Although obese patients have a larger blood volume, the vascularity of adipose tissue is lower than that of lean body mass, raising concern for over-anticoagulation when heparin dosing is calculated only with total body weight. Additionally, under-dosing is a significant concern as obese patients have been shown to be at increased risk of VTE recurrence. 21
Direct oral anticoagulants are not effective in all APS patients and should not be used routinely in these patients. Studies suggest a higher thrombotic risk in some APS patients treated with DOACs. 22 23
  Low-molecular-weight heparins (LMWHs) were recommended given warfarin failure. Enoxaparin is easy to administer and has a longer half-life, requiring once or twice daily dosing, and thus resulting in improved comfort for the patient, and better efficiency for the care team. Enoxaparin also has a low frequency of heparin-induced thrombocytopenia (HIT), which is a concern given the additional factors in dosage calculation. Given these factors, enoxaparin was determined to be the ideal next step for this patient.
Fondaparinux is used mainly for the treatment of HIT. 24 Although fondaparinux is effective in reducing episodes of thrombosis, there are little data about fondaparinux use in APS, 25 thus making fondaparinux a second choice after enoxaparin.
For patients that have a high INR, it is recommended to switch the anticoagulation therapy from warfarin to LMWH. 26 The patient was switched to enoxaparin and hydroxychloroquine was added for the thrombocytopenia. Although the anti-FXa assay can be used to monitor anticoagulation in patients who are taking LMWH, it is not generally used. 27 On a 30-day follow-up, there were no reported major bleeding complications or failures to anticoagulation.
  Answers: c, d, a, and c.

**Table 1:** Pertinent/elevated test results

Variables	Reference ranges	Test results				
GLU	70–99 (mg/dL)	123	102	104	102	102
BUN	6–24 (mg/dL)	42	41	37	36	31
CREAT	0.7–1.1 (mg/dL)	1.7	1.7	1.5	1.6	1.7
HGB	11.3–13.4 (g/dL)	11.6	11.5	11.8		
HCT	41–50 (%)	34.4	33.5	34.2		
PLT	150–400 (10 ^9^ /L)	94	100	101		
ALB	3.4–5.4 (g/dL)	3.0	3.3	3.1		
PT (patient was on warfarin)	11–15 (s)	18.1	27.8	35.7		22.3
INR		1.9	3.1	3.9		2.4
PTT	25–40 (s)	N/A	N/A	N/A	N/A	83
TSH	0.5–5.0 (mIU/L)	5.980				
CHOL	<200 (mg/dL)	213				
HDL	>45 (mg/dL)	39				

Abbreviations: ALB, albumin; BUN, blood urea nitrogen; CHOL, cholesterol; CREAT, creatinine; GLU, glucose; HCT, hematocrit; HDL, high-density lipoprotein; HGB, hemoglobin; INR, international normalized ratio; PT, prothrombin time; PTT, partial thromboplastin time; TSH, thyroid stimulating hormone.

## Discussion

SS is a rare medium-vessel vasculopathy which often presents with LRa and cerebrovascular disease. 14 It can be associated with APS, such as in this patient. The optimal management of patients with SS remains an unsolved issue and controlled trials have not yet been performed (see Table 2 ). Based on the presumed pathogenesis of SS, some researchers have recommended long-term anticoagulation for cerebral ischemic events. Antiplatelet and antithrombotic agents are used for secondary stroke prophylaxis. The most widely accepted treatment is anticoagulation with warfarin. High-dose warfarin with INR >3 is preferable in this situation. The benefits of warfarin are greater than the risks of bleeding. 28

**Table 2:** Sneddon syndrome treatment options according to level of evidence

Treatment options	Level of evidence
Antiplatelet therapy	I
Anticoagulant therapy: • Warfarin • DOACs • LMWH	II
Rituximab	III
Cyclophosphamide/azathioprine	IV
Thrombolytic therapy	IV

Abbreviations: DOACs, direct oral anticoagulants; LMWH, low-molecular-weight heparin.

Other therapeutic options such as azathioprine and cyclophosphamide do not appear to be effective. There is evidence suggesting that rituximab (RTX) could be beneficial for individuals with aPL-positive status. Several case reports describe the use of RTX in APS patients and one case report describes the use of RTX in a SS patient with good response and no relapses after 2 years of follow-up. 29 Even though there is no conclusive evidence, RTX could be considered as a treatment option for patients experiencing thrombotic relapses while on VKA and maintaining an adequate INR.

This patient's management was challenging because of the multiple comorbidities he presented and the presence of APL antibodies that was altering the INR. This elevation is thought to be reflective of the reaction of the aPL antibody with the thromboplastin used to measure the PT. 30 31

In these scenarios we need to rely on factor II and chromogenic factor X. These tests do not have alterations in the presence of APS antibodies, and their activity is reliable in patients who require alternative monitoring of warfarin. 32 Also, these factors are more important during clot formation than factor VII, on which INR is mainly based, and thus their reductions may better reflect anticoagulation. 33

Chromogenic assays are not dependent on thromboplastin or fibrinogen, thus bypassing in vitro interactions that could lead to a falsely elevated INR, thereby producing more reliable results than PT/INR and other clot-based assays in patients with APS. 34

Lately, it has been emphasized about the use of the Fiix prothrombin time to have more reliable and steady results while monitoring warfarin. The Fiix prothrombin time is a novel test (created by Fiix Diagnostics Ltd and commercialized by Hart Biologicals Ltd) that is only sensitive to reductions in FII and FX, in contrast to the standard PT/INR that is mainly affected by reductions of factor VII, thus stabilizing the VKA's effect. Although it can be a helpful laboratory tool, it is not yet available. In a single-center, randomized, controlled clinical trial by Onundarson et al, 1,156 patients were randomly assigned to either the Fiix-PT monitoring group or the PT monitoring group. Anticoagulation stability was improved with Fiix-PT monitoring as manifested by fewer tests, fewer dose adjustments, increased time in range, and less INR variability than reported with standard PT monitoring. 35

In the event of anticoagulation failure in patients on warfarin, there is not a standardized approach to it, nevertheless below we share things to consider when dealing with these patients. (see Table 3 ).

**Table 3:** Things to consider when dealing with anticoagulation failure in a patient on warfarin

*1. Confirm it is really a new thrombotic event.*	^●^ It is important that suspected treatment failure be a confirmed treatment failure and not a misdiagnosis of recurrent thrombotic event or due to nonadherence. ^●^ Falsely labeling a patient as having treatment failure leads to worry about underlying serious diagnoses and leads to an unnecessary escalation in anticoagulant therapy.
2. Check factor II or factor X.	^●^ Confirm medication adherence. ^●^ Time of therapeutic range (TTR) represents the percentage of time in which the INR remains in the 2.0 to 3.0 target range across time. ^●^ Check factor II and Xa
3. Adequate TTR and factor levels. Differential Dx.	^●^ Vasculitis 46 ^●^ Atheroembolic 47 ^●^ Malignancy 48 ^●^ Essential thrombocythemia 49 ^●^ Nonbacterial thrombotic endocarditis 50 ^●^ Decompression illness ^●^ Hyperviscosity syndrome

Abbreviation: INR, international normalized ratio.

First thing to do is to confirm that the patient is having a new thrombotic event. Is it really a new event or is it non adherence? Evaluate images, labs, and other diagnostic tests according to the event. This is an important first question to make in order to avoid unnecessary escalation in anticoagulant therapy. Also, it is important to differentiate between warfarin treatment failure (new thrombotic events despite an INR in therapeutic range) versus warfarin resistance (inability to maintain INR in therapeutic range).

TTR estimates the percentage of time a patient's INR is within the desired treatment range. Some authors define satisfactory INR control as a TTR equal or higher than 60%, 36 though this threshold is not universally agreed upon. However, TTR can be influenced by factors such as individual patient characteristics and or diseases, drug interactions, the frequency of INR monitoring, and treatment modifications.

Certain pathologic states like APS can alter INR, like in this case. Measurement of factor II or chromogenic factor X can help us in establishing a better control for these patients. However, recurrent thrombotic events can occur with normal levels of these factors through alternative mechanisms and pathways. An example of this can be, but is not limited to, a stroke due to small-vessel occlusion.

Due to the increasing utilization of the DOACs, it is important to consider certain aspects regarding treatment failure. Malabsorption is one of the main causes of DOAC treatment failure, especially in major gastrointestinal resections or bypasses. There is little evidence regarding the use of DOAC in these cases, but overall, its use is preferably avoided as first-line anticoagulation in patients who have undergone these types of surgeries until more data are available. 37 38 In addition, DOACs are not effective in APS. Studies suggest a higher thrombotic risk in APS patients treated with DOACs. 22 23

DOACs have fewer drug–drug interactions than warfarin. Common interactions are with drugs metabolized by the cytochrome P450 enzyme (CYP450) and the transporter permeability glycoprotein (P-gp). Every DOAC is a substrate for P-gp, that is why they are susceptible to drugs that induce or inhibit it. CYP3A4 is an important metabolizer for apixaban and rivaroxaban but not the other DOACs. 39

Strong CYP3A and P-gp inhibitors are itraconazole, ketoconazole, clarithromycin, lopinavir, indinavir, ritonavir, and telaprevir. P-gp inducers comprise carbamazepine, phenytoin, phenobarbital, rifampin, dexamethasone, tocilizumab, and St. John's Wort. 39

Patients with cancer have an increased risk of thrombotic events and they were historically managed with LMWHs. Recently, new studies demonstrated that DOACs are noninferior to LMWHs. Although there is still treatment failure with DOACs in cancer patients, recurrence rates of VTE are lower than those that were treated with dalteparin. In the ADAM VTE trial, recurrent VTE occurred in 0.7% of apixaban patients, compared to 6.3% of dalteparin patients. 40 In the HOKUSAI VTE trial, recurrent VTE occurred in 7.9% patients of the edoxaban group and 11.3% in the dalteparin group. 41 In addition, in the CARVAGGIO VTE trial, recurrent VTE occurred in 5.6% patients in the apixaban group and in 7.9% in the dalteparin group. 42

This patient would benefit from a factor II or a factor X level test to monitor long-term anticoagulation with warfarin, optimally using a chromogenic assay. However, these tests are expensive and not widely available to some clinicians. In situations where a patient experiences a repeated thrombotic event while on anticoagulation, there are several potential interventions to consider. One option for patients with a lower target INR is to increase the target range to 3 to 4 by administering high-intensity warfarin. 43 44 However, in a recent clinical trial involving individuals with aPL antibodies and ischemic stroke, it was observed that both aspirin (325 mg per day) and warfarin (target INR: 1.4–2.8) provided comparable antithrombotic benefits. 45 Conversely, for patients with an elevated INR, it is recommended to switch the anticoagulation therapy from warfarin to LMWH. 26 Due to our patient's elevated INR, the decision was made to transition from warfarin to LMWH as a preventive measure against future thromboembolic events. LMWH monitoring would be performed using the chromogenic anti FXa assay. Renally dosed LMWH was considered, contingent on close monitoring given his renal failure.
